# DNA methylation signatures of youth-onset type 2 diabetes and exposure to maternal diabetes

**DOI:** 10.1186/s13148-024-01675-1

**Published:** 2024-05-13

**Authors:** Ola E. Salama, Nikho Hizon, Melissa Del Vecchio, Kurt Kolsun, Mario A. Fonseca, David T. S. Lin, Oscar Urtatiz, Julia L. MacIsaac, Michael S. Kobor, Elizabeth A. C. Sellers, Vernon W. Dolinsky, Allison B. Dart, Meaghan J. Jones, Brandy A. Wicklow

**Affiliations:** 1 Department of Biochemistry and Medical Genetics, University of Manitoba, Winnipeg, MB Canada; 2 Diabetes Research Envision and Accomplished in Manitoba (DREAM) Theme of the Children’s Hospital Research Institute of Manitoba, Winnipeg, MB Canada; 3 Department of Pediatrics and Child Health, University of Manitoba, Winnipeg, MB Canada; 4 Department of Pharmacology and Therapeutics, University of Manitoba, Winnipeg, MB Canada; 5 Department of Medical Genetics, Faculty of Medicine, University of British Columbia, Vancouver, BC Canada; 6 Centre for Molecular Medicine and Therapeutics, Vancouver, BC Canada; 7 Edwin S.H. Leong Centre for Healthy Aging, University of British Columbia, Vancouver, BC Canada

**Keywords:** Diabetes in youth, DNA methylation, Type 2 diabetes, Epigenetics, In utero programing

## Abstract

**Objective:**

Youth-onset type 2 diabetes (T2D) is physiologically distinct from adult-onset, but it is not clear how the two diseases differ at a molecular level. In utero exposure to maternal type 2 diabetes (T2D) is known to be a specific risk factor for youth-onset T2D. DNA methylation (DNAm) changes associated with T2D but which differ between youth- and adult-onset might delineate the impacts of T2D development at different ages and could also determine the contribution of exposure to in utero diabetes.

**Methods:**

We performed an epigenome-wide analysis of DNAm on whole blood from 218 youth with T2D and 77 normoglycemic controls from the iCARE (improving renal Complications in Adolescents with type 2 diabetes through REsearch) cohort. Associations were tested using multiple linear regression models while adjusting for maternal diabetes, sex, age, BMI, smoking status, second-hand smoking exposure, cell-type proportions and genetic ancestry.

**Results:**

We identified 3830 differentially methylated sites associated with youth T2D onset, of which 3794 were moderately (adjusted *p*-value < 0.05 and effect size estimate > 0.01) associated and 36 were strongly (adjusted *p*-value < 0.05 and effect size estimate > 0.05) associated. A total of 3725 of these sites were not previously reported in the EWAS Atlas as associated with T2D, adult obesity or youth obesity. Moreover, three CpGs associated with youth-onset T2D in the *PFKFB3* gene were also associated with maternal T2D exposure (FDR < 0.05 and effect size > 0.01). This is the first study to link *PFKFB3* and T2D in youth.

**Conclusion:**

Our findings support that T2D in youth has different impacts on DNAm than adult-onset, and suggests that changes in DNAm could provide an important link between in utero exposure to maternal diabetes and the onset of T2D.

**Supplementary Information:**

The online version contains supplementary material available at 10.1186/s13148-024-01675-1.

## Introduction

In recent decades, there has been a steady increase in the number of youth newly diagnosed with type 2 diabetes (T2D) across many countries [1]. These youth face higher rates of complications including diabetic kidney disease, the most prevalent complication, and other micro-, and macrovascular complications, implying differences in the broader health impacts of T2D early in life compared to later [2, 3]. While adult-onset T2D is characterized by the gradual progression of insulin resistance and *β*-cell dysfunction [4], youth-onset T2D is characterized by the rapid onset of hyperglycemia and rapid deterioration in *β* cell function, further indicating that there may be a different set of risk factors and mechanisms involved [1]. At present, these mechanisms are poorly understood and genetic variants only explain ~ 5–10% of T2D heritability [5, 6], suggesting that factors in the environment likely contribute to the development of T2D and its associated complications [7]. An established risk factor for youth-onset T2D is exposure to diabetes during pregnancy [8, 9]. Dysglycemia during pregnancy is associated with offspring obesity [10], insulin resistance [10] and cardiovascular diseases [11]. However, the mechanistic link between in utero exposure to diabetes and the increased risk of developing youth-onset T2D in the offspring is not clear. A deeper understanding of the molecular mechanisms underlying youth-onset T2D are needed for the development of environmental and intervention programs that improve clinical outcomes and prevent the onset of complications.

Altered DNA methylation (DNAm) associated with T2D in a peripheral tissue is one way we may be able to better understand how youth-onset T2D differs from adult-onset. It is also a potential mechanism linking early life exposures such as maternal T2D to long-term health outcomes in the offspring. DNAm is an epigenetic modification that involves the addition of a methyl group to the 5’ position of cytosine [12]. DNAm is associated with gene expression and can modify the accessibility of transcription factors to the promoter region of genes [13]. Typically, the methylation of a promoter is associated with transcriptional silencing, while demethylation is associated with transcriptional activation [13, 14]. DNAm patterns are associated with a number of environmental exposures, including prenatal exposure to maternal obesity [15] and dysglycemia [16].

To date, there have been no epigenome-wide investigations specifically involving youth with T2D despite the unique pathophysiology, natural history, and significant increase in incidence over the last decade. Since the initial description of youth-onset T2D [17], there have been investigations into the unique aspects of pathophysiology [4] and risk factors associated with the development of T2D [2, 18]. However, we do not know how youth-onset T2D specifically affects DNAm, nor do we know how much of the DNAm signature of youth-onset T2D is influenced by in utero exposure to maternal diabetes. Therefore, our objective was to identify altered DNAm in youth with T2D from the improving renal Complications in Adolescents with type 2 diabetes through REsearch (iCARE) cohort, and determine whether any of those differences were associated with in utero diabetes exposure using an epigenome-wide approach. We identified 36 differentially methylated sites that were strongly associated with T2D, only three of which had been previously associated with T2D in adults as reported in the EWAS Atlas. We also found three sites that were associated with both youth-onset T2D and exposure to intrauterine maternal diabetes. Enrichment analysis provided insight into the biological pathways and processes associated with differentially methylated sites, and we found that DNAm changes in whole blood are enriched in insulin signaling pathways. The findings of this study could contribute new knowledge about the unique molecular mechanisms underlying the health impacts of youth-onset T2D and aid in the development of interventions.

## Materials and methods

### iCARE cohort

This study was a cross-sectional analysis of a subset of the iCARE (improving renal Complications in Adolescents with type 2 diabetes through REsearch) longitudinal cohort study. The study was approved by the University of Manitoba Research Ethics Board (HS13255/ B2021:079). The iCARE cohort is Canada’s largest prospective observational cohort study of youth living with T2D, who were between 10 and 18 years of age at the baseline research visit. The cohort was established to identify the biological, psychological and social determinants of kidney complications in children diagnosed with T2D, and the study methodology has previously been published [19]. Initially established in Manitoba, Canada, seven additional Canadian centers were added in 2016. In brief, youth were eligible if they had a clinical diagnosis of type 2 diabetes based on Canadian national clinical practice guidelines [20], which are aligned with the American Diabetes Association Criteria, and were negative for autoantibodies. Youth were excluded from the study if they had another form of diabetes (medication induced, surgical), were currently treated with medications which were expected to affect metabolism (steroids, antipsychotics), were pregnant, or had a history of cancer or immunosuppressive therapy. Whole blood samples were collected from enrolled and consenting iCARE participants at the baseline visit of the study. Approximately 62% of the entire iCARE cohort consented to and had whole blood for epigenetic analysis collected. Whole blood DNAm was assessed from 319 individuals using the Illumina Infinium Human Methylation EPIC BeadChip after DNA extraction using QIAamp DNA blood minikit (Qiagen, USA) and bisulfite conversion using the Zymo EZDNA methylation kit (Zymo, USA).

### Quality control and normalization

Red and Green channel intensity values from 319 samples and 1,051,815 probes from the two color Illumina Infinium Human EPIC BeadChip microarray were parsed from IDAT files into R programming language using the *minfi* package [21]. Detection p-values were calculated for each probe by comparing total signal from methylated and unmethylated probes for each position to the background signal level from negative control probes. No samples were excluded by mean detection *p*-value < 0.05. Probe intensity levels (866,091 CpG loci) were normalized for background signal with normal-exponential out-of-band normalization using *preprocessNoob* from *minfi*. A total of 2504 probes were excluded from analysis due to low detection *p*-values (*p*-value < 0.01 in any sample). Next, 78,355 genomic probes with bead counts < 3 in at least three samples, 7651 CpG loci in the sex chromosomes, 27,071 CpG loci commonly affected by a known single nucleotide polymorphism (SNP), and 36,636 CpG loci with cross-reactive probes were excluded in that order leaving 705,247 CpG loci for analysis. Cross-reactive probes were obtained by combining the findings of Chen et al. 2014 [22], McCartney et al. 2016 [23], and Pidsley et al. 2016 [24]. Probe-type variance was corrected for using *BMIQ* from the *watermelon* package [25, 26]. One sample was repeated 4 times as technical replicates to track during normalization steps—at the end of normalization, the replicates had correlations > 0.99. Of these four replicates, one was randomly chosen and the rest were excluded from downstream analysis, leaving 316 samples and 705,247 CpG loci. Beta values were calculated as described [27].

### Cell-type deconvolution

Cell-type proportions of whole blood from each participant were estimated using a flow-sorted blood tissue reference set from *ExperimentHub* [28] and *estimateCellCounts2* function from the *FlowSorted.Blood.EPIC* package. Cell-type proportion predictions are closed compositional data which is not compatible with linear regressions and so we used the *compositions* R package to transform cell-type proportion information with isometric log-ratio using the *clr()* function from which robust principal components were extracted using *prcomp()* [29]. These orthogonal measures were included in the linear regression to account for cell-type proportion differences in DNAm.

### Genotyping

Genotype data were measured on Infinium Global Screening Array-24. Genotype data was exported into MAP and PED files for analysis in *plink* [30]*.* 23,387 variants with overlapping coordinates were excluded. 14,584 variants with call rates lower than 95% were excluded. 348,033 variants with minor allele frequencies less than 0.01 were excluded. 389 variants not following Hardy–Weinberg (*p*-value < 0.00001) in control samples were excluded. All samples had call rates above 95%. 301,090 variants in linkage disequilibrium, as calculated by window size of 50 and *r*^2^ threshold of 0.2, were excluded. Five principal components were extracted from the remaining 117,896 variants using EIGENSTRAT [31] to obtain continuous, orthogonal measures of genetic variance to be included in the linear regression model.

### EWAS statistical analyses

To identify differentially methylated CpGs associated with youth-onset type 2 diabetes, we performed linear regression for each of the 704,709 CpG sites with youth-onset type 2 diabetes status as the main variable and adjustment for maternal diabetes, age, sex, BMI, smoking, second-hand smoking, four cell-type principal components (PCs), five genotype PCs, and batch effects (BeadChip number and BeadChip position) using the *limma* package [32]. 295 individuals had complete data for all of those covariates and were included in the analysis. Benjamini–Hochberg [33] was used to correct for multiple tests with significance set at FDR < 0.05, and we used two effect size cut-offs: a less stringent 1% and a more stringent 5%. Bias and inflation of epigenome-wide association analysis test statistics were addressed by a Bayesian estimation of the empirical null distribution as implemented in *bacon* [34]. Briefly, a three-component normal mixture is fit into the test statistic distribution, one to capture the empirical null distribution, and other two capture true positive and negative associations. Differentially methylated regions (DMR) were identified using the *combp()* function from the *missMethyl* R package, and significant DMRs were identified using a spatially adjusted *p*-value < 0.05 and a minimum number of probes per DMR of two [35].

To test the association of youth-onset type 2 diabetes-associated CpG sites to maternal diabetes exposure (defined as the presence of type 2 diabetes or gestational diabetes during pregnancy), we performed a linear regression for each of the 3830 CpG sites significantly associated with youth-onset type 2 diabetes at the lower cutoff of FDR < 0.05 and effect size > 1% with maternal diabetes exposure as the main variable and adjusted for age, sex, BMI, smoking, second-hand smoking, four cell-type proportions, five genotype PCs, and batch effects (BeadChip number and BeadChip position) using the *limma* package [32].

### Sensitivity analysis

To ensure that the results are not stratified by ethnicity or smoking status, which were unbalanced between groups in our study, we performed sensitivity analyses for each of those variables. In both cases, we repeated the same linear regression used for the main analysis in a subset of individuals; [1] exclusively the First Nation subset to determine whether our findings were consistent in a single ethnic group, and [2] on the subset of the cohort who smoked to determine whether our findings were consistent in a group without differences in smoking status. Participants were given the opportunity to list up to three ethnicities, all participants that self-identified as either First Nation or Metis were included in the First Nations subset, and smoking was recorded by self-report. We calculated the 95% confidence interval for each site in both analyses, and if the effect size plus or minus the confidence intervals were consistent between the full model and the subset, we identified that site as consistent in the sensitivity analysis.

### Enrichment analyses

To identify biological processes and traits associated with the identified genes, we performed enrichment analysis. The background 704,709 CpGs were mapped to Entrez gene IDs using the *missMethyl* R package [35]. The 3830 differentially methylated sites and 1802 DMR sites were tested for enrichment for all gene sets in all terms in the Gene Ontology database [36] and all pathways in the KEGG database [37]. A Wallenius’ noncentral hypergeometric test was applied to account for the bias caused by CpGs mapping to multiple genes and genes containing multiple CpGs. Gene ontology results of biological processes were reported for each of the two tests, and the top 10 gene sets were ordered by increasing *p*-value.

### Epigenetic age analysis

Epigenetic age was calculated from unnormalized data using the established eAge calculator [38]. We calculate epigenetic age acceleration as the residuals of a regression between epigenetic and chronological ages, and tested for group differences using t tests.

## Results

### The improving renal complications in adolescents with type 2 diabetes through research (iCARE) cohort

316 youth participants (ages 12–24, mean 15 years of age) in the iCARE cohort were recruited from nine pediatric sites across Canada [19]. For the current study, 218 iCARE participants diagnosed with T2D according to the Diabetes Canada criteria [39] prior to 18 years of age under current treatment at any of the nine pediatric sites were identified along with 77 youth matched for ethnicity and BMI, all of whom had complete data (Table 1). The cohort is predominantly indigenous youth (85%). Youth with T2D had higher BMI (mean WHO BMI Z-score difference = 0.5, *p*-value = 3.47 × 10^–4^) and had a higher exposure rate to maternal diabetes (61% vs. 34%, *p*-value = 2.47 × 10^–4^) compared with controls.

**Table 1:** Characteristics of iCARE cohort participants

	Total (N = 295)	Case (N = 218)	Control (N = 77)	*P*-value*
*Sex*				
Female	190 (64%)	150 (69%)	40 (52%)	6.45 × 10^–3^
Male	105 (36%)	68 (31%)	37 (48%)	
*Ethnicity*				
Indigenous	251 (85%)	181 (83%)	70 (91%)	7.67 × 10^–2^
Non-Indigenous	44 (15%)	37 (17%)	7 (9%)	
*Age (years)*				
Mean (SD)	15 (± 3.0)	16 (± 2.8)	14 (± 3.2)	1.31 × 10^–5^
*WHO BMI (Z-score)*				
Mean (SD)	2.3 (± 1.0)	2.4 (± 0.93)	1.9 (± 1.2)	3.47 × 10^–4^
*Smoking status*				
No	242 (82%)	169 (78%)	73 (95%)	2.28 × 10^–3^
Occasional	12 (4%)	11 (5%)	1 (1%)	
Yes	41 (14%)	38 (17%)	3 (4%)	
*Maternal diabetes status*				
Gestational Diabetes	55 (19%)	42 (19%)	13 (17%)	2.47 × 10^–4^
Normoglycemic	135 (46%)	84 (39%)	51 (66%)	
Pre-Gestational Diabetes (T2D)	105 (36%)	92 (42%)	13 (17%)	
*Diabetes duration (years)*				
Mean (SD)	3.2 (± 2.7)	3.2 (± 2.7)	NA (± NA)	

^*^For age and BMI, t-test was used. Chi-square was used for the rest of the variables

### Youth with youth-onset T2D displayed altered DNA methylation patterns

**Fig. 1:**
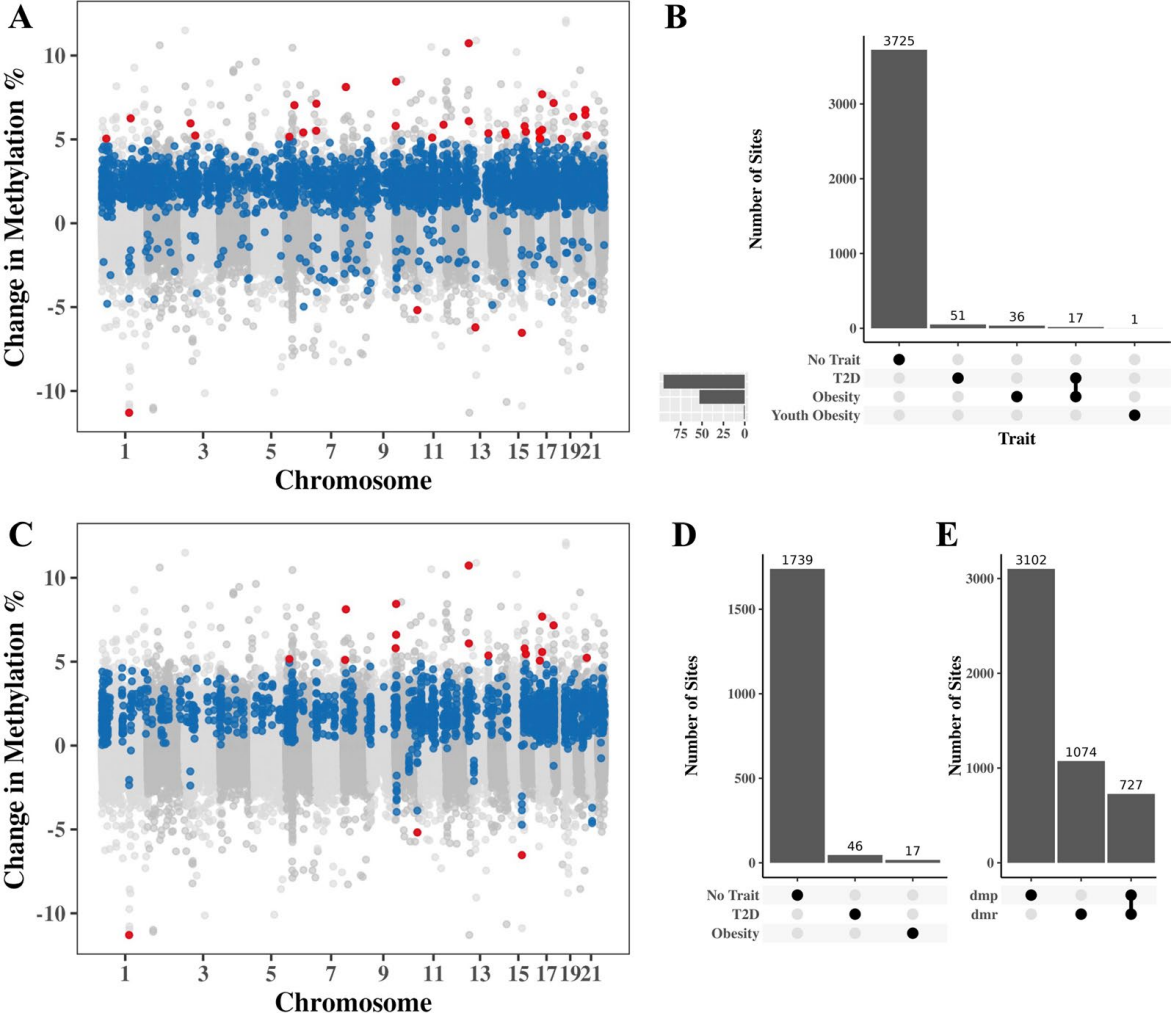
3830 CpG sites are differentially DNAm methylate in PBMCs of participants with youth-onset type 2 diabetes. **A** Difference in DNAm (y-axis) against the genomic location (x-axis) in 704,709 sites across the human genome in youth with T2D and controls. Sites that did not have significant association were colored gray. 3794 CpG sites (blue) were found to have moderate association to childhood-onset type 2 diabetes (adjusted *p*-value < 0.05 and effect size estimate ≥ 0.01). 36 CpG sites (red) were found to be strongly associated with youth-onset type 2 diabetes (adjusted *p*-value < 0.05 and effect size estimate ≥ 0.05). Associations were tested using multiple linear regression models from *limma*, using own diabetes as the main variable while adjusting for maternal diabetes, sex, age, BMI, smoking status, second-hand smoking exposure, cell-type proportions, and genetic ancestry. **B** Overlaps between sites identified in this study and those previously associated with disease per EWAS Atlas. Out of 3830 differentially methylated sites, 3725 sites had not previously been significantly associated with any disease, while the rest were associated with at least one of T2D, obesity, or child obesity. **C** 1802 individual sites were identified as part of DMRs, and **D** 1739 of these sites were not previously statistically associated with any disease while the rest were associated with T2D or obesity. **E** There were 728 common sites between individual site analysis and DMR analysis

To identify DNAm changes specific to youth-onset T2D, DNAm levels at 704,709 sites across the human genome were compared between youth with T2D and controls. Comparison of DNAm levels was done using multiple linear regression using presence of youth T2D as the main variable with adjustment for maternal diabetes, age, sex, BMI, smoking, second-hand smoking, cell-type proportions, genetic ancestry, and batch effects (BeadChip number and BeadChip position) (Additional file 1: Fig. 1). 3830 differentially methylated sites were identified between youth with youth-onset T2D and controls at FDR < 0.05 and ≥ 1% difference in DNAm (Fig. 1A, Additional file 2: Supplementary Table 1), and 36 of these sites had difference in DNAm  ≥ 5% between the groups (Table 2, Additional file 1: Supplementary Fig. 2). Epigenome-wide association analyses are prone to bias and inflation due to the larger number of expected true associations, and we calculated an inflation factor of 1.30 (Additional file 1: Supplementary Fig. 1). We used the BACON package to estimate the empirical null distribution and account for bias and inflation. This analysis identified 39,493 differentially methylated sites at FDR < 0.05 and ≥ 1% change in DNAm , with DNAm changes ≥ 5% in 41 of these sites (Additional file  1: Supplementary Fig. 2). These results include all 3830 differentially methylated sites prior to bias correction, so we continued with our non-BACON-corrected analyses. We also performed differentially methylated region analysis (DMR), and identified 516 DMRs containing 1802 sites in total (Fig. 1C). 17 of these sites had a difference in DNAm methylation ≥ 5% between the groups (Additional file  3: Supplementary Table 2). There were 728 common sites between individual CpG analysis and DMR analysis (Fig. 1D).

**Table 2:** Thirty-six DNAm sites associated (FDR < 0.05 and effect size > 0.05) with youth-onset type 2 diabetes in peripheral blood (n = 295)

CpG	Chr	Position	UCSC RefGene Name	Gene Position	Relation to Island	Adjusted p-value	Effect Size*	EWAS Atlas Previous Association	mQTL
cg20150640	1	23,697,656	C1orf213;ZNF436	3'UTR;Body;1stExon;TSS1500	S_Shore	4.10E−02	5.04E−02	No	Yes
cg19693031	1	145,441,552	TXNIP	3'UTR	OpenSea	1.50E−10	-1.13E−01	Walaszczyk et al*.*, 2018, Soriano-Tárraga et al*.*, 2016, Kulkarni et al*.*, 2015	No
cg12538074	1	153,716,514	INTS3	Body	OpenSea	4.15E−04	6.25E−02	No	No
cg04915160	3	32,087,236			OpenSea	4.79E−02	5.95E−02	No	No
cg25799109	3	57,102,900	ARHGEF3;SPATA12	5'UTR	OpenSea	1.91E−02	5.23E−02	No	No
cg17758563	6	16,323,116	ATXN1	Body	N_Shelf	1.68E−05	5.15E−02	No	No
cg07710211	6	42,900,123	CNPY3	Body	S_Shelf	5.31E−03	7.04E−02	No	Yes
cg16809457	6	90,399,677	MDN1	Body	OpenSea	1.30E−04	5.41E−02	No	No
cg11991942	7	220,911	FAM20C	Body	S_Shore	3.89E−02	5.51E−02	No	Yes
cg04816311	7	1,066,650	C7orf50	Body	N_Shore	5.50E−06	7.13E−02	Meeks et al. 2019, Cardona et al*.*, 2019	No
cg25332918	7	158,766,061			N_Shore	2.11E−02	8.12E−02	*Weng *et al*., 2018*	Yes
cg18581998	9	136,335,884	CACFD1	3'UTR	OpenSea	1.10E−03	5.80E−02	No	No
cg06132598	9	138,899,436	NACC2	3'UTR	OpenSea	1.46E−02	8.44E−02	No	No
cg07216976	10	116,581,248	FAM160B1	TSS1500	N_Shore	6.02E−03	-5.18E−02	No	No
cg15068842	11	60,677,810			N_Shelf	1.39E−03	5.10E−02	No	No
cg10052504	11	121,243,886			OpenSea	2.20E−02	5.87E−02	No	Yes
cg26996569	12	121,829,743			OpenSea	1.19E−03	1.07E−01	No	No
cg24288706	12	122,287,927	HPD	Body	OpenSea	4.72E−02	6.09E−02	No	No
cg21860329	13	42,265,546	VWA8	Body	OpenSea	4.54E−04	-6.21E−02	No	No
cg26548185	13	111,464,743			Island	6.78E−03	5.37E−02	No	No
cg12944343	14	93,698,416			OpenSea	4.43E−02	5.43E−02	No	Yes
cg07107661	14	97,877,297			OpenSea	1.42E−02	5.26E−02	No	No
cg10149159	15	80,189,934	MTHFS	TSS1500;Body	S_Shore	2.19E−02	-6.53E−02	No	No
cg09530217	15	101,728,353	CHSY1	Body	OpenSea	5.90E−03	5.45E−02	No	No
cg00658411	16	4,467,558	CORO7	TSS1500	S_Shore	3.42E−02	5.79E−02	No	Yes
cg01928516	17	2,208,377	SMG6;SRR	TSS1500;5'UTR	S_Shore	1.47E−05	5.45E−02	No	No
cg17146917	17	4,674,194	TM4SF5	TSS1500	OpenSea	4.40E−03	5.06E−02	No	No
cg25164957	17	7,236,973			S_Shelf	4.15E−02	5.02E−02	No	No
cg25393494	17	17,109,936	PLD6	TSS1500	Island	1.83E−05	7.69E−02	No	Yes
cg24578857	17	17,110,207	PLD6	TSS1500	Island	5.01E−03	5.58E−02	No	Yes
cg15244183	18	11,143	LOC102723376	TSS1500	N_Shore	3.58E−02	7.17E−02	No	No
cg21240131	18	43,715,467			OpenSea	2.40E−02	5.03E−02	No	No
cg06381803	19	46,119,475	EML2	Body	Island	2.69E−03	6.35E−02	No	No
cg18704705	20	47,364,640	PREX1	Body	OpenSea	3.91E−02	6.46E−02	No	No
cg13619283	20	47,364,643	PREX1	Body	OpenSea	3.49E−02	6.75E−02	No	No
cg14929076	20	55,041,057			N_Shelf	1.12E−03	5.23E−02	No	Yes

^*^Control—T2D

To determine which of our findings had previously been associated with T2D, we leveraged the EWAS Atlas database [40]. The CpG IDs of the 3830 CpGs with > 1% DNAm change were input to the atlas. We found that 3725 sites were not previously reported as associated with T2D, adult obesity, or youth obesity (Fig. 1B). CpGs that had > 5% DNAm change and were not associated with the “type 2 diabetes” trait in the EWAS Atlas were labelled as not previously associated (Table 2). For sites that had difference in DNAm  ≥ 5% between the groups and were previously associated with T2D, adult obesity or youth obesity, we examined chromatin information of lymphoblastoid cells from GM12878 using the UcscTrack() function from the gviz R package (Additional file 1: Supplementary Fig. 4).

Since shared genetic variation between mother and child could increase risk of T2D and also alter DNAm patterns, we tested whether the identified 36 individual sites and 17 DMRs with high effect sizes were associated with methylation trait quantitative loci (mQTL), using the mQTLdb [41]. Ten out of the 36 individual sites were associated with an mQTL (*p-value* < 1E−14 and effect size > 0.02%), and none of DMR sites were associated with an mQTL.

### Sensitivity tests

**Fig. 2:**
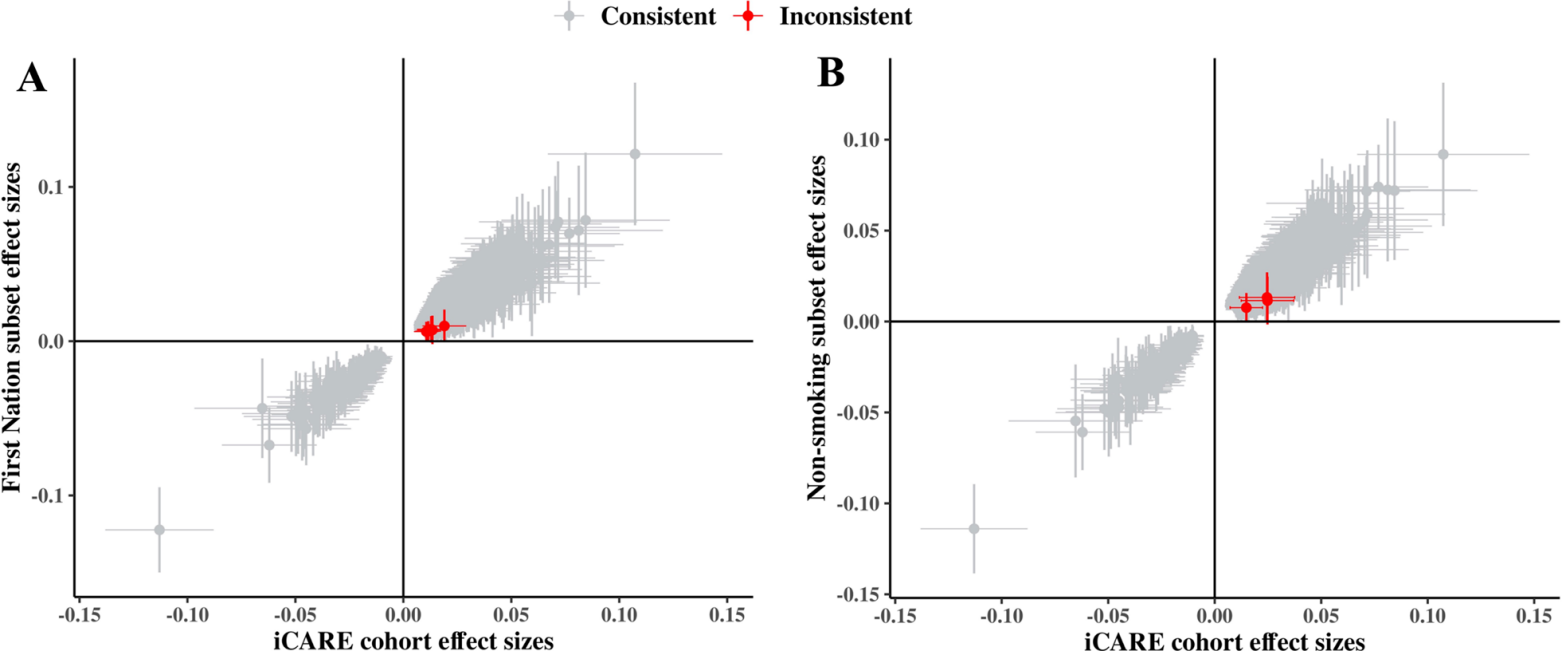
Epigenome-wide association sensitivity test of ethnicity and smoking status in the iCARE cohort. Red crosses represent CpGs with inconsistent direction of change between the whole cohort and the subset cohort. **A** Only five out of 3830 CpGs (FDR < 0.05 and effect size > 0.01) were found to have an inconsistent direction of change between the whole cohort and First Nation subset EWAS. **B** Only three out of 3830 CpGs (FDR < 0.05 and effect size > 0.01) were found to have inconsistent direction of change between the whole cohort and non-smoking subset

Given that both ethnicity and cigarette smoking are known to have strong effects on DNAm levels and the iCARE cohort includes multiple ethnic groups, as well as a mixture of smokers and non-smokers, we performed sub-analyses to ensure the results were not influenced by ethnicity or smoking status. The same 704,709 DNAm sites were analyzed using the same regression and covariates in two subsets: 251 participants from the self-declared First Nation subset (Additional file 4: Supplementary Table 3) or 242 non-smokers. Effect size measures from this regression were compared with effect size measures from the whole cohort analysis, and if the 95% confidence intervals were in the same direction in the sub-analysis as in the full cohort, we considered the site to be consistent (Fig. 2A). Out of 3830, only five had an inconsistent direction of change in DNAm in the ethnicity sub-analysis, and three differentially methylated sites had inconsistent direction of change in DNAm in the smoking sub-analysis (Fig. 2B), indicating that our findings are robust to these potential confounders.

### Enrichment analysis

The 3830 differentially methylated sites and 1802 DMR sites were investigated for enrichment for gene ontology sets and pathways in the KEGG using a Wallenius’ noncentral hypergeometric test. The top results in the biological processes highlighted DNAm changes in sugar and protein catabolic and metabolic processes (Additional file 5: Supplementary Table 4 and Additional file 6: Supplementary Table 5). Similar results were observed in the KEGG pathway enrichment, glycerophospholipid, riboflavin, and nicotinate and nicotinamide metabolism (Additional file 7: Supplementary Table 6 and Additional file 8: Supplementary Table 7). Interestingly, in our individual sites analysis, DNAm methylation changes are also enriched in the insulin signaling pathway (unadjusted *p*-value = 7.03 × 10^–3^) and vasopressin-regulated water reabsorption pathway (unadjusted *p*-value = 7.43 × 10^–3^); however, after adjusting for multiple testing, only the Apelin signaling pathway remained significant (Additional file 7: Supplementary Table 6).

### Epigenetic age analyses

As a measure of overall change in DNAm which may not be identified at individual sites or DMRs, we tested whether youth-onset T2D or exposure to maternal diabetes accelerated biological aging as measured by two epigenetic clocks. Neither own diabetes nor exposure to intrauterine maternal diabetes altered epigenetic age at the time of blood draw using either the Horvath or SkinBlood epigenetic clocks (Additional file 1: Supplementary Fig. 5). 

### Association of childhood-onset T2D DNAm patterns with exposure to intrauterine maternal diabetes

**Fig. 3:**
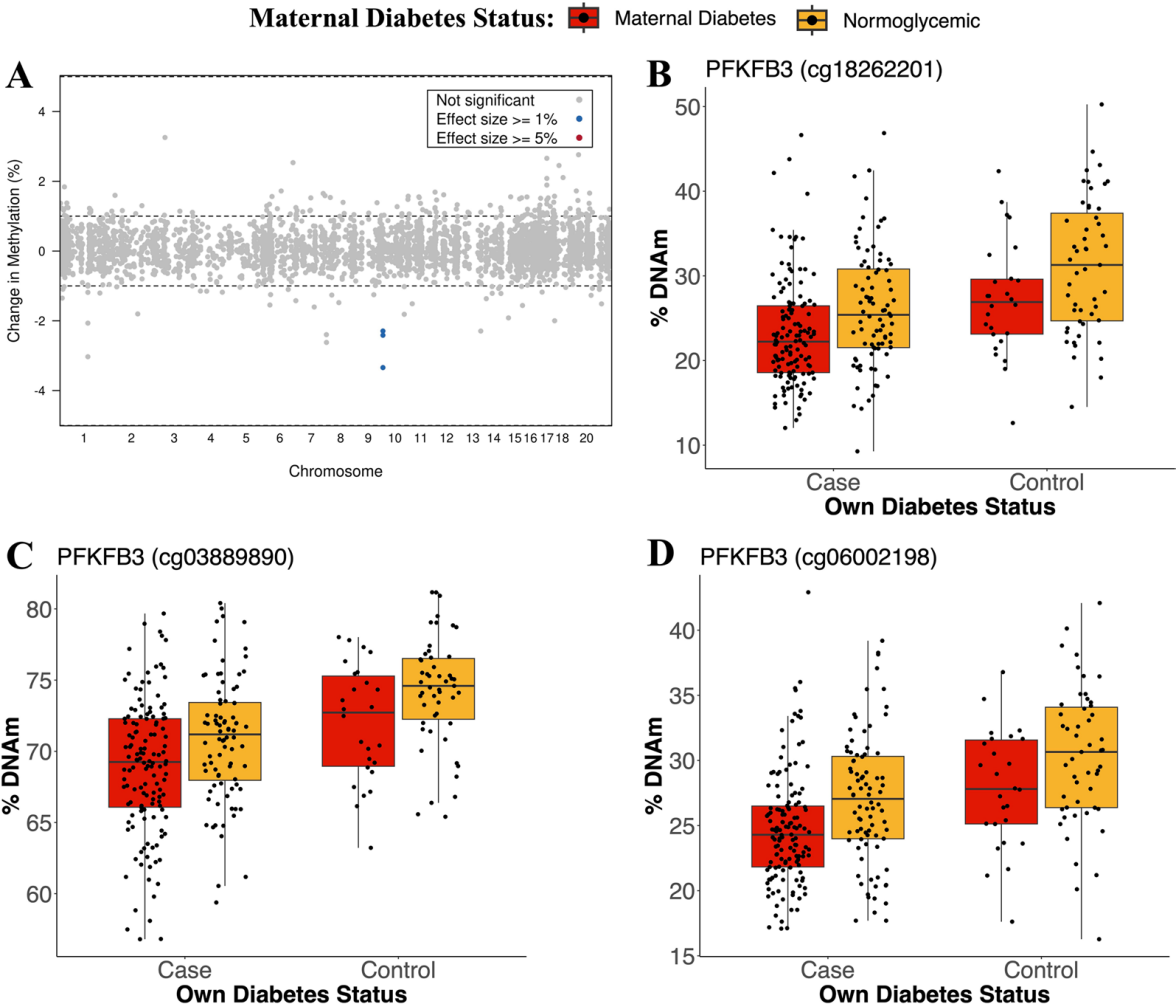
Three CpGs associated with youth-onset T2D are also associated with exposure to maternal diabetes. **A** 3 CpGs were found moderately associated with maternal diabetes exposure (FDR < 0.05 and effect size ≥ 0.01). **B**–**D** Exposure to intrauterine maternal diabetes and own diabetes had additive effects on DNAm at the 3 CpG sites

Exposure to intrauterine maternal diabetes has been previously identified as a risk factor for youth-onset T2D, and previously was associated with DNAm changes. Therefore, we investigated whether any of the sites we identified as associated with youth-onset T2D were also associated with exposure to intrauterine maternal diabetes. The cohort was split into two groups: youths exposed to intrauterine maternal diabetes (including gestational and pre-gestational diabetes) and youths exposed to a normoglycemic intrauterine environment. DNAm methylation at the 3830 differentially methylated sites associated with youth-onset T2D were compared between the two groups using a multiple linear regression with adjustment for age, sex, smoking, second-hand smoking, four cell-type PCs, five genetic PCs, and batch effects (Additional file 1: Supplementary Fig. 6). Three sites were associated with exposure to intrauterine maternal diabetes (FDR < 0.05 and ≥ 1% change in DNAm, Table 3, Fig. 3A) in the *PFKB3* gene, at 1500 bp upstream (-2.41% change in DNAm, FDR = 1.65 × 10^–2^), 200 bp upstream (-2.29% change in DNAm, FDR = 1.65 × 10^–2^), and within the gene body (-3.34% change in DNAm, FDR = 1.65 × 10^–2^) the *PFKFB3* gene (Fig. 3B–D). None of these sites have previously been associated with an mQTL (Table 3).

**Table 3:** Altered DNAm in youth with type 2 diabetes associated with intrauterine maternal diabetes exposure

CpG	Chromosome	Position	UCSC RefGene name	Gene position	Relation to Island	Adjusted p-value	Change in DNAm (%)	mQTL
cg06002198	10	6,187,994	PFKFB3;PFKFB3	TSS1500;Body	S_Shore	0.0165	− 2.41	No
cg03889890	10	6,188,223	PFKFB3;PFKFB3	TSS200;Body	S_Shore	0.0165	− 2.30	No
cg18262201	10	6,187,854	PFKFB3	Body	Island	0.0165	− 3.34	No

## Discussion

To the best of our knowledge, this is the first epigenome-wide study to investigate associations between DNAm and youth-onset T2D with the goal of isolating changes associated with the unique pathophysiology of this disease, and to see whether maternal diabetes exposure in utero primes these diabetes-associated DNAm differences. We identified 3830 individual sites and 516 differentially methylated regions in peripheral blood with > 1% difference in DNAm between youth with and without T2D, of which 36 sites and 17 DMRs displayed > 5% difference. Of these, three sites in *PFKFB3* were also associated with exposure to intrauterine maternal diabetes, potentially implicating DNAm changes with the transmission of T2D risk by maternal diabetes [9]. The majority of the DNAm changes we identified had not previously been reported as associated T2D and could provide insight into the aggressiveness of complications and early onset of youth-onset T2D. Overall, our findings suggested that DNAm could be a biomarker for youth-onset T2D, and that maternal diabetes exposure in combination with own diabetes demonstrated synergistic effects on DNAm at *PFKFB3*.

Comparing our results with previous findings from epigenome-wide association analyses on adult-onset T2D recorded in the EWAS Atlas [40], the majority of our sites were not associated with T2D, obesity or youth obesity. This provides additional credence to the concept that youth-onset T2D is different from adult-onset T2D [1]. In the 36 sites that had > 5% DNAm change, two were previously associated with adult-onset T2D; *TXNIP* [42–44], and a predicted open reading frame *C7orf50* [45, 46]. By contrast, another study that also examined *TXNIP* and *C7or50* found no association between DNAm at those genes and T2D [47], but this could be due to limited sample size (101 individuals with T2D in the discovery stage of the study and 66 individuals in their replication stage). Note that we did not perform a meta-analysis of existing T2D studies to determine whether any of our sites demonstrated similar effect sizes but did not meet statistical significance in other studies. However, given that the EWAS Atlas reported only 5.5% (2/36) of our large effect sites and 2.7% (105/3830) of our medium effect size sites as previously statistically associated with T2D or T2D-related traits across 23 studies, our results support that the effects on blood DNAm of youth-onset T2D are very different from adult-onset.

Three differentially methylated sites in youth-onset T2D were also associated with exposure to intrauterine maternal diabetes in a dose dependent manner, all in *PFKFB3*. These sites showed decreased DNAm in youth of mothers with T2D which was even lower in youth who had T2D themselves, suggesting that in utero exposure to diabetes might change the DNAm profile for those sites which are then further altered if the youth develop T2D. Pancreatic *β*-cells are non-regenerative and when injured, they activate the HIF1α/PFKFB3 injury regeneration metabolic pathway [48]. This pathway ensures the survival of the injured pancreatic cells, but forces the cells to rely on glycolysis for ATP production, making them less responsive to glucose for insulin production. PFKFB3 protein is predominantly expressed in endothelial cells and plays an important role in glycolysis [49], and expression of *PFKFB3* was increased in *β*-cell nuclei of rats and humans with T2D [48]. In a previous study, *PFKFB3* had been linked previously to type 1 diabetes; compared to nondiabetic, type 1 diabetes individuals had a larger proportion of *β*-cells that expressed *PFKFB3* [50]. Combined with our results, these findings suggest that in utero exposure to maternal diabetes could affect the expression of *PFKFB3*, either in *β*-cells themselves or in other tissues, which in return affects the responsiveness to glucose. We acknowledge that blood-based DNAm may not always directly reflect DNAm methylation in other cell and tissue systems such as the *β*-cell [51]. Given the inaccessibility of *β*-cell tissue as a biomarker, blood-based DNAm can nonetheless still provide useful information about systemic changes related to conditions such as diabetes that could be utilized as a biomarker. This study, as far as we know, is the first to link *PFKFB3* to T2D in youth.

Due to the cross-sectional nature of our study, we are unable to distinguish between DNAm changes that lead to youth-onset T2D and DNAm changes caused by youth-onset T2D. Another limitation to our study is having unbalanced groups; however, our sensitivity tests showed that ethnicity and smoking status did not drive our association results. Our study also did not disentangle the differential effects of gestational diabetes mellitus (GDM) versus maternal pre-gestational T2D on DNAm. We have previously shown that maternal pre-gestational T2D confers a greater risk of youth-onset T2D than GDM [9]. Future studies could be adequately powered to determine whether there is a differential impact of GDM vs maternal T2D on DNAm in youth living with T2D. Future prospective and longitudinal studies are also needed to test for the specific association between DNAm changes such as we have identified and development of T2D complications.

In Canada, First Nations youth account for a higher relative number of T2D diagnoses compared to the general population [52]. The majority of individuals in the iCARE cohort study are of First Nations background [19]. We showed here that maternal diabetes alone has long lasting influences on DNAm methylation patterns in youth living with T2D. T2D in youth is associated with poverty and lower socioeconomic status [53]. Colonization has profoundly altered the health of indigenous peoples resulting in the biological manifestation of chronic disease. Many additional environmental exposures such as purposeful starvation, nutrition, stress and trauma have effects on the epigenome that merit consideration in understanding the development of T2D in First Nations youth [54–56].

Overall, our results show that DNAm patterns associated with youth-onset T2D differ greatly from adult-onset. We also showed that in utero exposure to maternal diabetes is associated with DNAm changes that are linked to youth-onset T2D. This study provides some insight to the unique molecular pathways which are disrupted by youth-onset T2D and could potentially aid in the development of intervention programs aimed at preventing the serious and unique health effects of early development of T2D.

### Supplementary Information

**Additional file 1. Figure 1:** (A) Linear regression results of epigenome-wide analysis of associations between youth-onset type 2 diabetes and whole blood DNA methylation. (B) QQ plot of p-values depicting presence of slight inflation (lambda = 1.33).

**Additional file 2. Table 1:** Altered DNA methylation in youth with youth-onset T2D at FDR < 0.05 and effect size >= 1%.

**Additional file 3. Table 2:** DMR sites associated with youth with youth-onset T2D at FDR < 0.05 and effect size >= 1%.

**Additional file 4. Table 3:** Characteristics of First Nation participants in the iCARE cohort.

**Additional file 5. Table 4:** Biological processes enrichment analysis for individual CpG analysis.

**Additional file 6. Table 5:** Biological processes enrichment analysis for DMR analysis.

**Additional file 7. Table 6:** KEGG pathway enrichment analysis for individual CpG analysis.

**Additional file 8. Table 7:** KEGG pathway enrichment analysis for DMR analysis.

## Data Availability

Data sharing is not applicable to this article to protect study participant privacy. This is a primarily indigenous cohort and OCAP principles apply.
