# Supplementary material for: DNA methylation signatures of youth-onset type 2 diabetes and exposure to maternal diabetes
Source: Clin Epigenetics. 2024 May 13;16:65. doi: 10.1186/s13148-024-01675-1 (PMC11092083; doi:10.1186/s13148-024-01675-1)
Supplement: Supplementary file 1 — Additional file 1. Figure 1: (A) Linear regression results of epigenome-wide analysis of associations between youth-onset type 2 diabetes and whole blood DNA methylation. (B) QQ plot of p-values depicting presence of slight inflation (lambda = 1.33). [file 13148_2024_1675_MOESM1_ESM.docx]

**Supplementary Figures:**

**
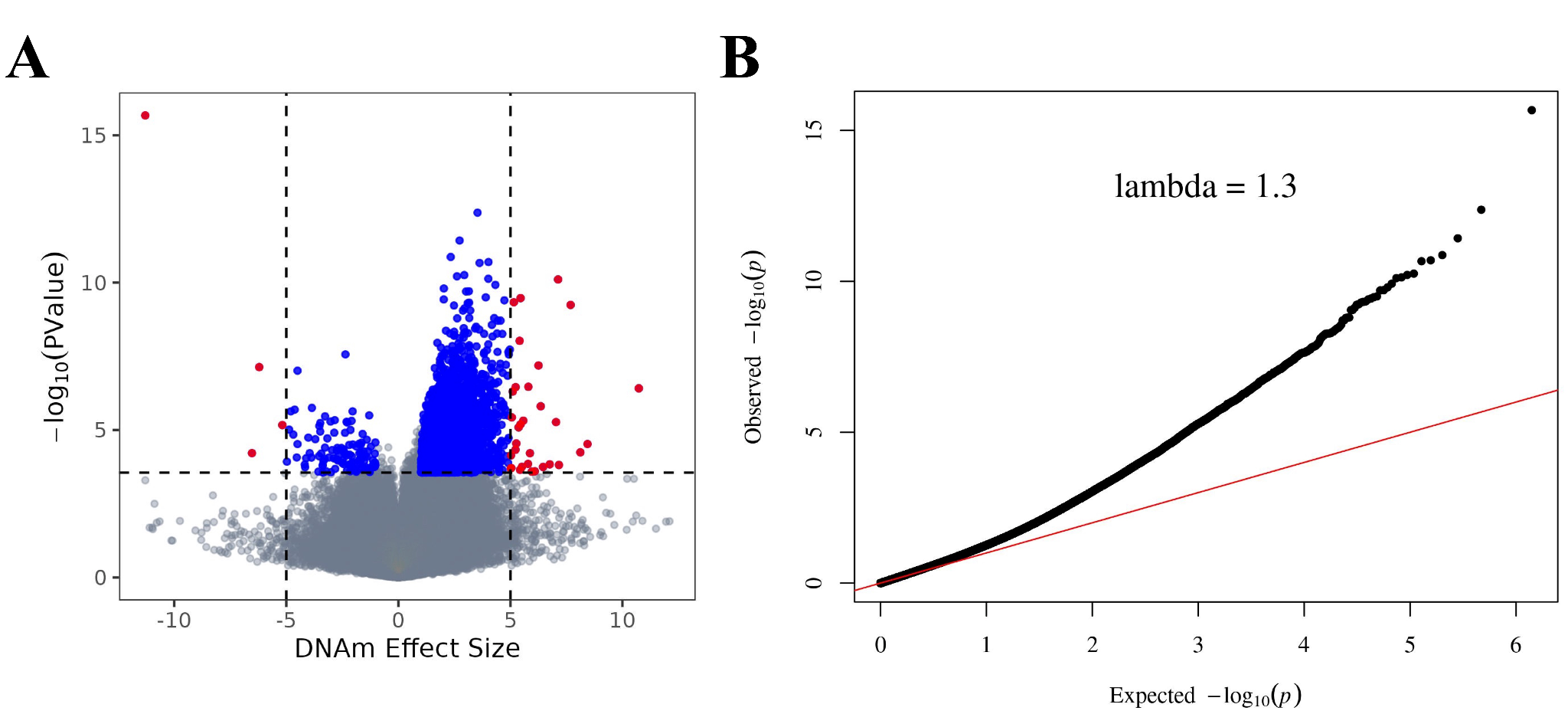
Supplementary Figure 1:** (A) Linear regression results of epigenome-wide analysis of associations between youth-onset type 2 diabetes and whole blood DNA methylation. (B) QQ plot of p-values depicting presence of slight inflation (lambda = 1.33).

**
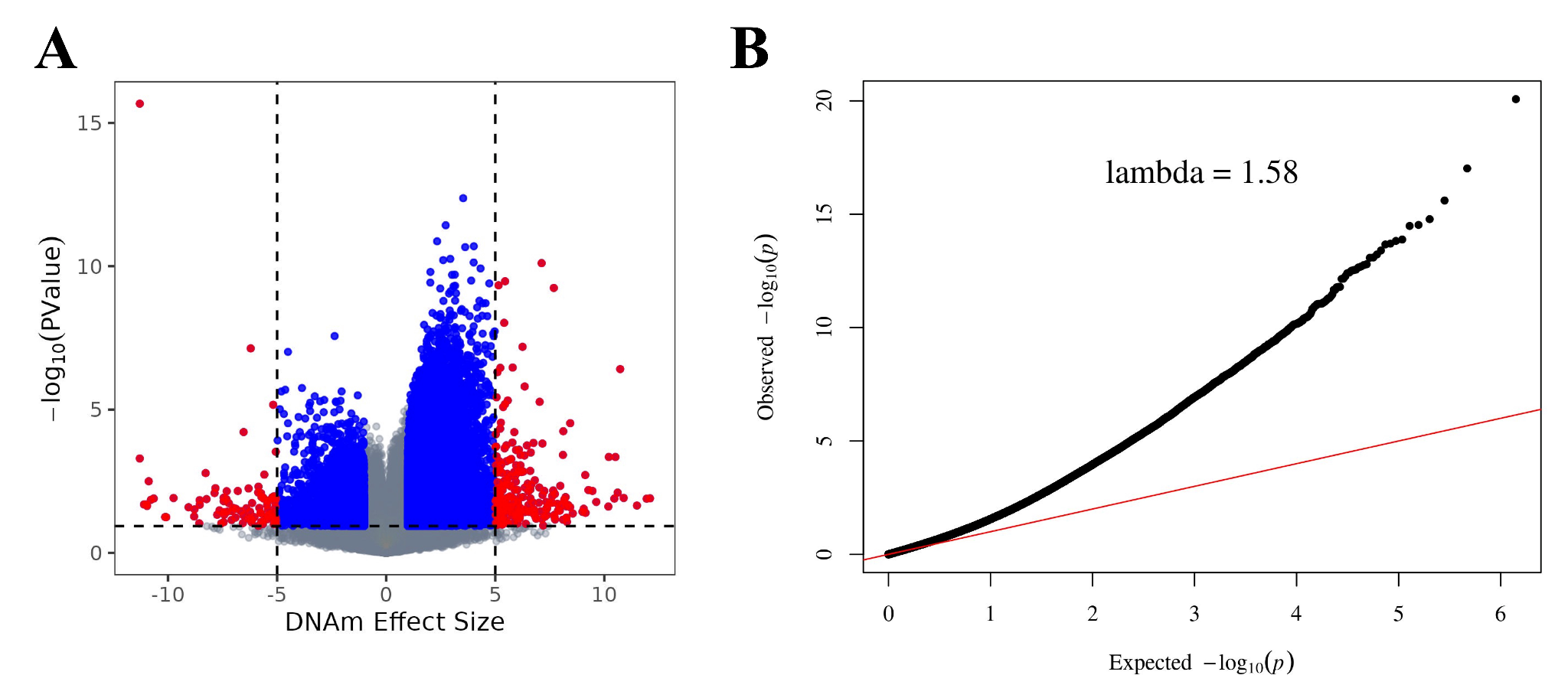
Supplementary Figure 2:** (A) Bacon correction for bias and inflation on the test statistics of epigenome-wide analysis of associations between youth-onset type 2 diabetes and whole blood DNA methylation. (B) Bacon-corrected p-values depicting a slight increase in inflation (lambda = 1.59).

**
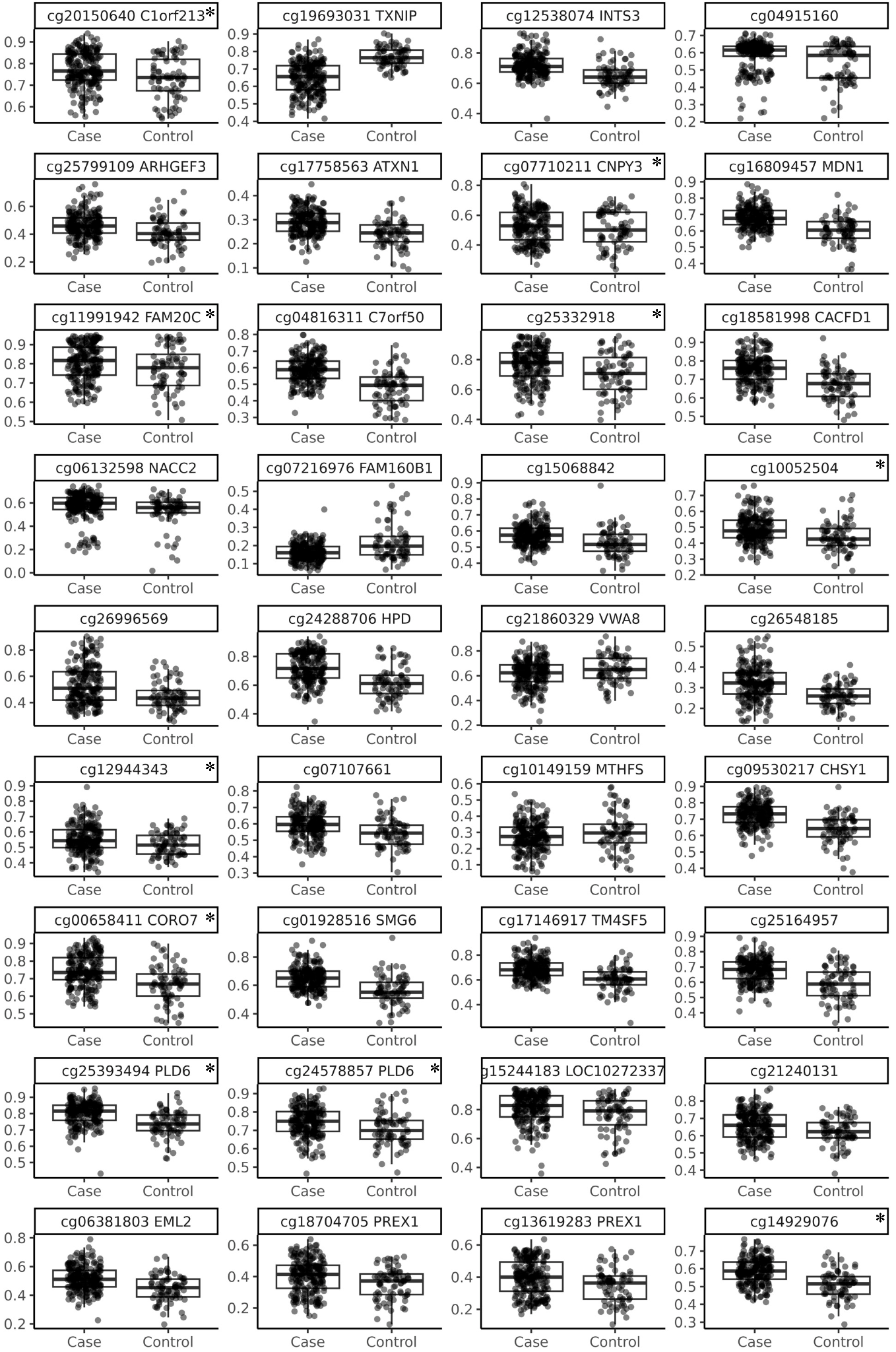
**

**Supplementary Figure 3: Comparison of DNA methylation differences identified levels between youth with T2D and controls.** The DNA methylation level of the 36 CpG sites that were strongly associated with diabetes were different between the two groups (FDR < 0.05 and > 5% change in methylation). An asterisk denotes association with an mQTL as identified in the mQTLdb.

**
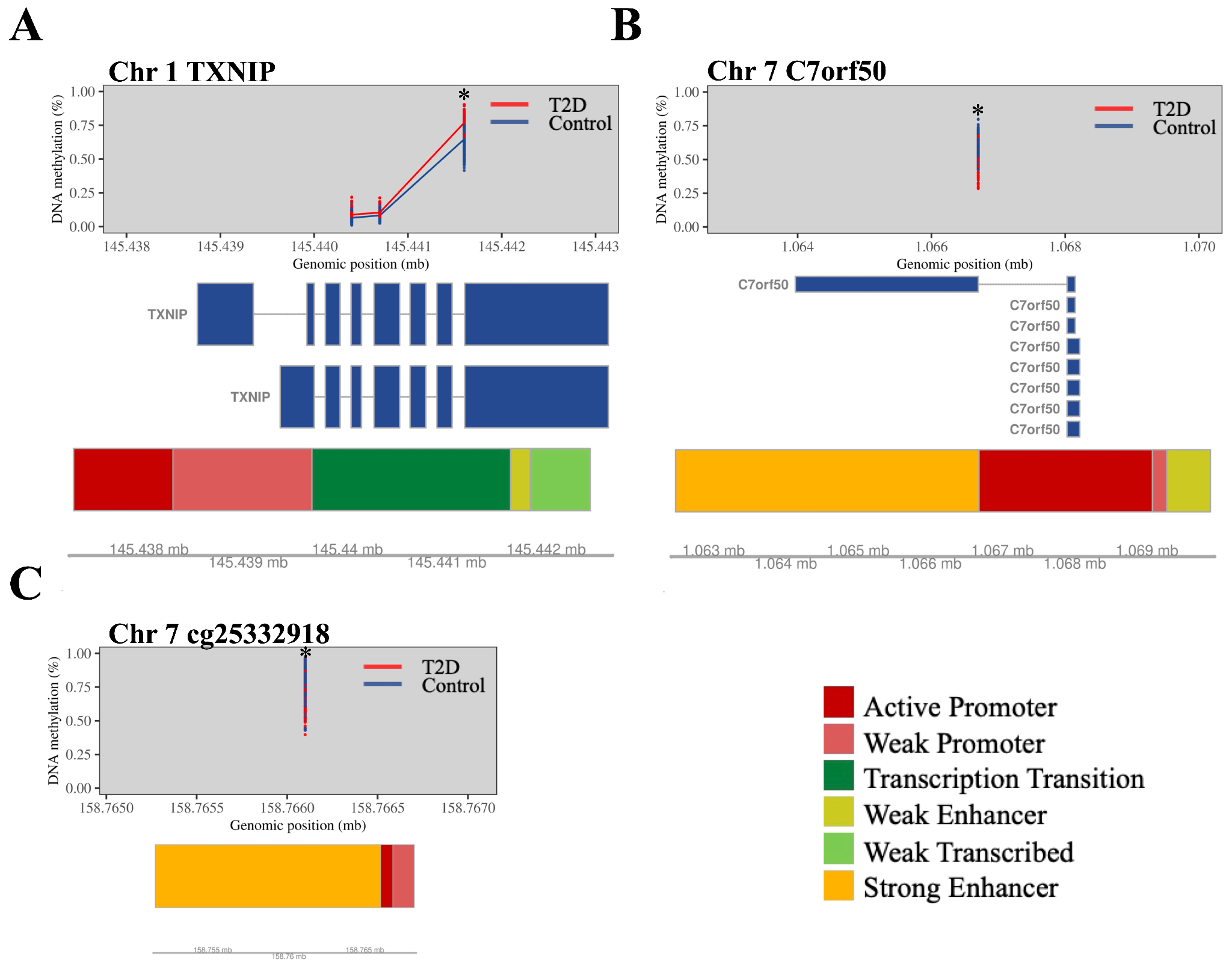
Supplementary Figure 4: DNA methylation and chromatin state.** DNA methylation at CpGs Located in **(A)** TXNIP within Chr1: 145,438,240-145,442,406, **(B)** C7orf50 within Chr 7: 1,064,112-1,069,343, and **(C)** cg25332918 within Chr 7: 158,765,061-158,767,061 are shown. Annotation and chromatin state were obtained from the University of California Santa Cruz using the UcscTrack() function from the Gviz R package. Chromatin states of GM12878 blood tissue cell line were used to obtain the chromatin state. Asterisks denote sites that were strongly associated with diabetes (FDR < 0.05 and > 5% change in methylation).

**Supplementary Figure 5: Epigenetic age is not accelerated by exposure to maternal in utero diabetes or own diabetes.** Epigenetic age acceleration as measured by the Horvath (top) and SkinBlood (bottom) clocks was not different between T2D cases and controls nor by maternal diabetes status.
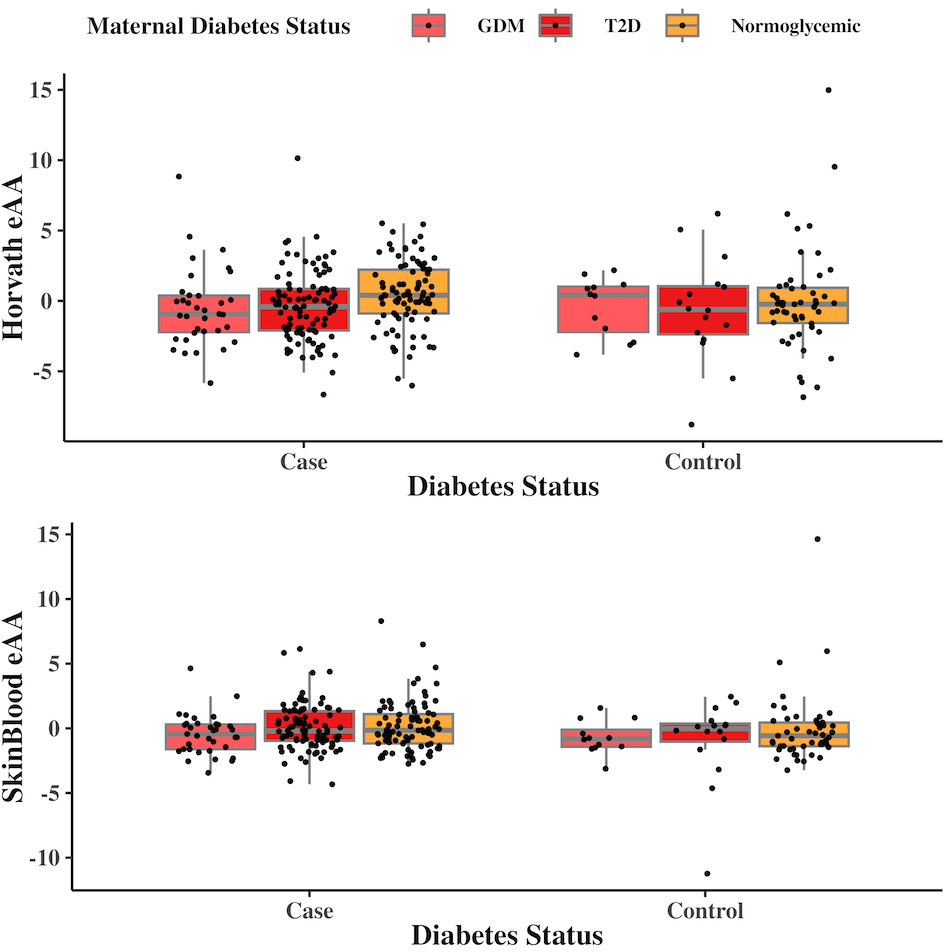


**
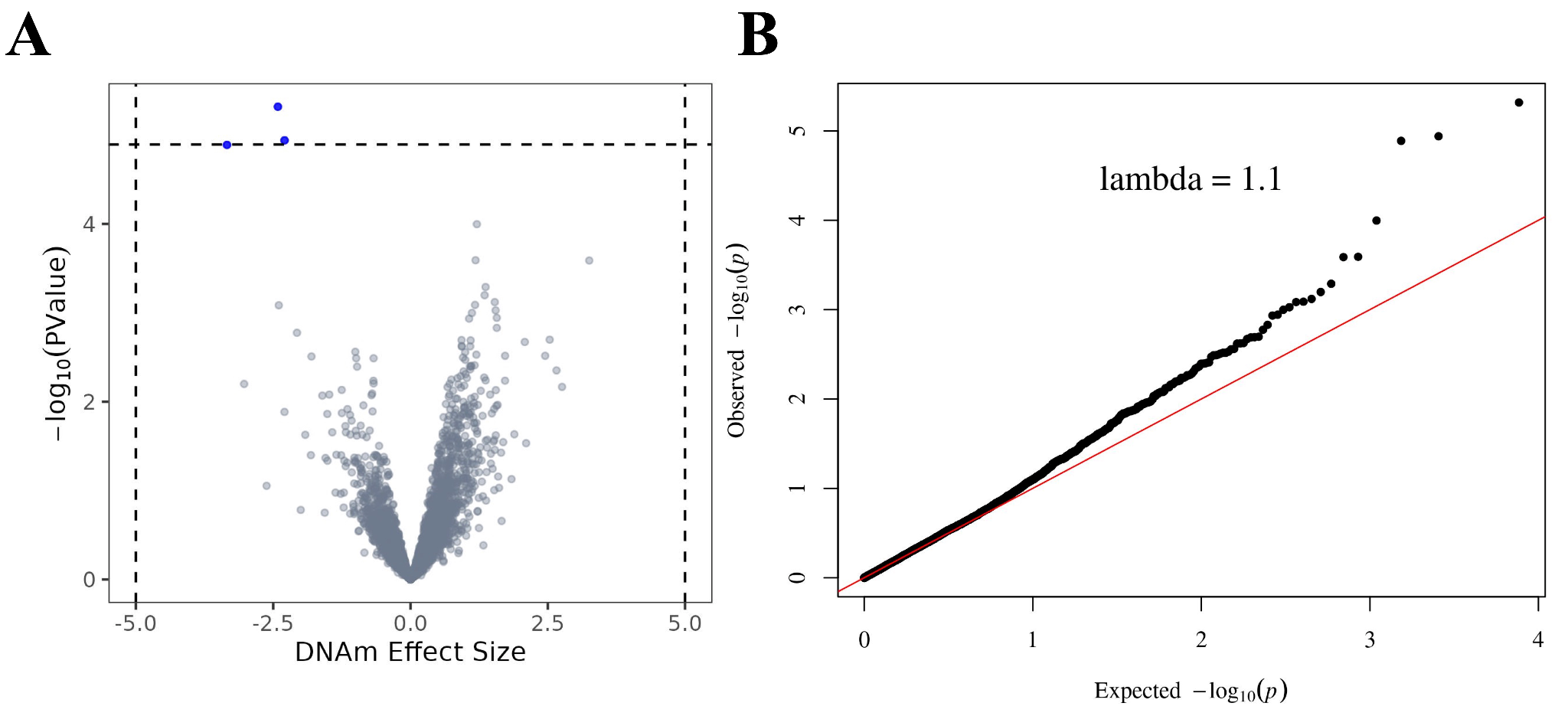
**

**Supplementary Figure 6:** (A) Linear regression results of epigenome-wide analysis of associations between in-utero exposure to maternal diabetes and whole blood DNA methylation. (B) QQ plot of p-values depicting presence of slight inflation (lambda = 1.10).
