# Supplementary material for: DNA methylation signatures of youth-onset type 2 diabetes and exposure to maternal diabetes
Source: Clin Epigenetics. 2024 May 13;16:65. doi: 10.1186/s13148-024-01675-1 (PMC11092083; doi:10.1186/s13148-024-01675-1)
Supplement: Supplementary file 4 — Additional file 4. Table 3: Characteristics of First Nation participants in the iCARE cohort. [file 13148_2024_1675_MOESM4_ESM.docx]

**Supplementary Table 3: Characteristics of First Nation participants in the iCARE cohort.**

|  | **Total (N=251)** | **Control (N=70)** | **Case (N=181)** |
| --- | --- | --- | --- |
| **Sex** |  |  |  |
| Female | 157 (63 %) | 33 (47 %) | 124 (69 %) |
| Male | 94 (37 %) | 37 (53 %) | 57 (31 %) |
| **Age (years)** |  |  |  |
| Mean (SD) | 15 (± 3.0) | 14 (± 3.1) | 16 (± 2.8) |
| **BMI (Z-score)** |  |  |  |
| Mean (SD) | 3.3 (± 0.27) | 3.3 (± 0.27) | 3.3 (± 0.28) |
| **Smoking** |  |  |  |
| Yes | 37 (15 %) | 3 (4 %) | 34 (19 %) |
| Occasional | 12 (5 %) | 1 (1 %) | 11 (6 %) |
| No | 202 (80 %) | 66 (94 %) | 136 (75 %) |
| **Maternal Diabetes Status** |  |  |  |
| Normoglycemic | 111 (44 %) | 46 (66 %) | 65 (36 %) |
| Gestational Diabetes | 50 (20 %) | 12 (17 %) | 38 (21 %) |
| Pre-Gestational Diabetes (T2D) | 90 (36 %) | 12 (17 %) | 78 (43 %) |
| **Diabetes Duration (years)** |  |  |  |
| Mean (SD) | 3.2 (± 2.6) | NA | 3.2 (± 2.6) |
